# Comparative Quantification of Diffusion Tensor Tractography Using Automated Whole Brain MRI Tractography for Intracranial Tumor Surgery: Technical Note

**DOI:** 10.7759/cureus.25546

**Published:** 2022-05-31

**Authors:** Cindy Alms, Chikezie I Eseonu

**Affiliations:** 1 Neurological Surgery, University of Pittsburgh Medical Center (UPMC) Central Pennsylvania, Harrisburg, USA

**Keywords:** whole brain tractography, diffusion tensor imaging, diffusion tensor tractography, cylindrical retraction, brain tumor

## Abstract

With the improvement of diffusion tensor imaging (DTI) and algorithms, diffusion tensor tractography (DTT) may provide quantitative information on white matter tracts (WMT) that may help quantitatively assess WMT integrity and distortion, which may help with correlations of neurologic function or prognosis. This manuscript is the first to describe a technical method for quantitative analysis of clinically relevant white matter tracts during intracranial tumor surgery.

The authors quantitatively analyzed relevant proximal WMT, pre and postoperatively, in a patient undergoing cranial surgery using DTT software to evaluate fractional anisotropy (FA), mean diffusivity (MD), radial diffusivity (RD), axial diffusivity (AD), geodesic anisotropy (GA), tract count, and tract volume. A method was then established to formulate quantitative comparisons between pre and postoperative WMT.

Quantitative assessment of the corticospinal and optic radiation tracts revealed significant increases in the FA, GA, and tract count in the corticospinal and optic radiations postoperatively (p<.0001). MD, RD, and AD were found to be significantly diminished postoperatively (p<.0001). The postoperative optic radiations showed diminished volume as a result of damage to the tract pathway.

To conclude, the utilization of white matter tractography provides a technical advancement that allows for quantitative comparative assessments of white matter tracts, which could assess the degree of brain changes following tumor surgery.

## Introduction

Minimally invasive intracranial surgery using tubular retraction, endoscopy, and small craniotomies have often been used as methods to preserve eloquent cortical regions and deep subcortical white matter while addressing cranial pathology. With growing knowledge about the function of the brain, developing new means to evaluate and assess brain function and white matter changes becomes increasingly important. White matter tractography is a non-invasive method used to visualize the tracts of white matter in the brain [1]. Diffusion tensor imaging (DTI) uses the directional diffusion of molecules of water to calculate the white matter tracts (WMTs) in the brain [2]. Using this method, major white matter tracts can then be identified and visualized. Diffusion tensor tractography (DTT) uses the information of DTI to create a three-dimensional white matter fiber tract of the brain and has been used to qualitatively evaluate alterations, disruptions, or displacements of the white matter tracts caused by intracranial lesions [3].

Although qualitative data from DTT has helped with preoperative and intraoperative surgical planning, quantitative assessments of the white matter tracts have not been practically used in the operative setting [4-12]. Quantitative data generated from DTT scans could potentially give rise to more evaluation criteria and prognostic indicators that can help with assessing and predicting patient outcomes in the preoperative and postoperative settings. This study aims to propose a technique for using quantitative measurements to evaluate white matter tractography and evaluate the feasibility of quantitative DTI use in the operative neurosurgical setting.

## Technical report

Clinical presentation

**Figure 1:**
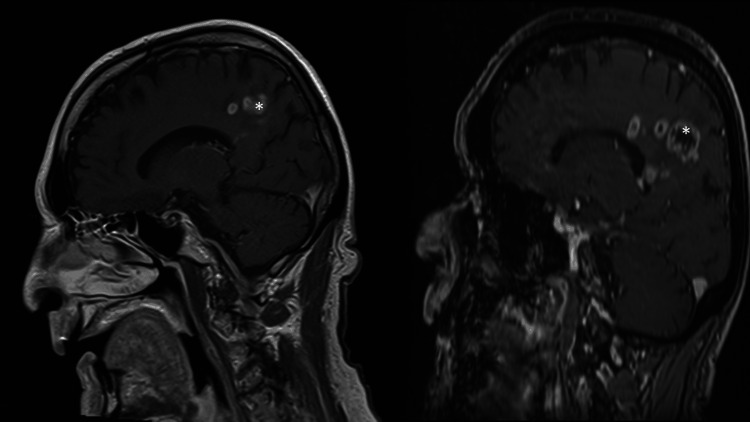
Sagittal MRI T1 with contrast scans of the initial presentation of the patient with right frontoparietal intraparenchymal lesions (LEFT), which showed significant growth over a one month time frame (RIGHT) Tumor labeled with (*)

The patient was an 80-year-old male presenting with headaches, who was found on MRI brain imaging to have multiple right frontoparietal subcortical heterogeneously enhancing masses (2.5 x 1.7 cm, large, enhancing mass with extension into the frontal subcortex with four additional satellite lesions, < 0.7 cm, along the splenium of the corpus callosum). Metastatic workup of the chest, abdomen, and pelvis was unrevealing for a primary tumor. The patient was briefly lost to follow-up, and a repeat MRI done one month later showed significant growth of the lesions (Figure 1). The preoperative motor strength of the left arm was 4+/5 strength and 5/5 in the left leg, with no visual field cuts noted on examination. An excisional biopsy of the largest, more superficial lesion was subsequently planned using diffusion tensor imaging (DTI) tractography.

Methods

MR/DT Imaging

MR imaging was performed using a 1.5T Toshiba Titan MRI (Toshiba Corporation, Minato, Tokyo). Pre and postoperative MRI scans were obtained. T1-weighted pre and post-contrast, as well as T2-weighted scans, were acquired with 1 mm-2 mm slice thickness with 0.0 mm gap/spacing between slices, with no overlap between the slices. The DTI imaging was acquired in 2-3 mm thick slices with a minimum of 20 gradient directions. A 0.0 mm gap/spacing was between slices with no overlap. The DTI data were then processed and converted into three-dimensional whole-brain tractography images using Modus Plan™ software (Synaptive, Toronto, Ontario). The Modus Plan™ was also auto-segmented into individual white matter tracts. All volumetric calculations were conducted with the Modus Plan™ software.

Procedure

**Figure 2:**
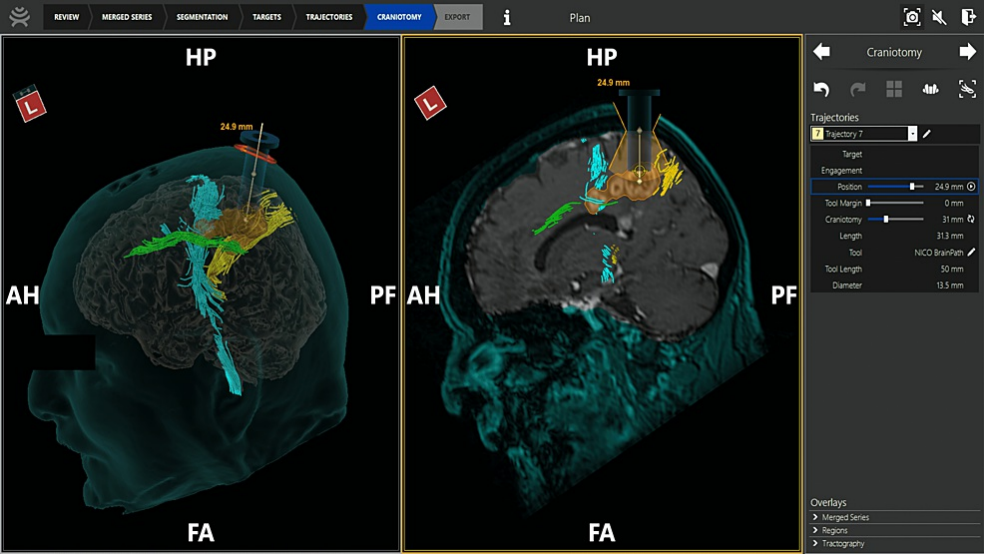
Preoperative planning using the NICO BrainPath cylindrical retraction system The large frontoparietal target lesion is highlighted on imaging, without including the deeper corpus callosal lesions that were not surgical targets. The segmented white matter tracts are visualized. (Light blue: Corticospinal tract, Yellow: Optic pathway, Green: Cingulum). HP- head posterior, AH- anterior head, PF-posterior foot, FA- foot anterior.

Preoperative imaging was obtained, as described above, 48 hours prior to surgery. The patient was brought into the operative room and positioned in a supine, slightly flexed position using a Mayfield clamp. The head was then registered to the preoperative MRI imaging using CranialMap 3.0 and NAV3i (Stryker, USA) for stereotactic neuronavigation. A small parietal craniotomy was made with a small corticectomy in the superior parietal lobule gyrus, and the BrainPath (Nico Corporation, Indianapolis) cylindrical retraction device was inserted into the brain toward the lesion of interest to conduct the tumor resection (Figure 2).

Somatosensory evoked potentials and electroencephalogram were monitored during the operation. Postoperative imaging was obtained on postoperative operative day 1. DTI imaging from both pre and postoperative scans was processed using Modus Plan™ software (Synaptive, Toronto, Ontario) as well as for visualizing tractography measurements.

Tractography Analysis

Modus Plan™ software (Synaptive, Toronto, Ontario) was used to quantify fractional anisotropy (FA), mean diffusivity (MD), radial diffusivity (RD), axial diffusivity (AD), geodesic anisotropy (GA), tract count, and tract volume. Tract evaluations for the corticospinal tract and optic radiations were conducted. To compare pre vs postoperative tractography, the respective quantified value was normalized using the median value of the contralateral tract (which had normal anatomy) to account for motion artifact between the scans (i.e. [preop right FA median/(preop right FA median + preop left FA median)] was compared to the [postop right FA median/(postop right FA + postop left FA median)]. For the cortical spinal tract quantifications, 3881 preoperative samples, generated by Modus Plan™, were taken for each preoperative quantitative metric, and 6411 postoperative samples were taken for each quantitative measure, respectively. For the optic radiation quantifications, 3484 preoperative samples were taken for each preoperative quantitative measure, and 3081 postoperative samples were taken for each quantitative measure, respectively. Statistical analysis was performed using a T-test on these samples. The institutional review board approved this study.

Results

Clinically, the patient’s intraoperative course was unremarkable. Given the small craniotomy used for BrainPath, motor mapping was not utilized in this case. Tumor debulking was conducted up until the area where the tumor was seen to be invading the corticospinal tract based on the intraoperative tractography imaging. Postoperatively, the patient regained 5/5 strength in his left arm at his two-week postoperative follow-up visit. Visual fields remained full throughout the postoperative course. Final pathology revealed a glioblastoma (IDH-wildtype, TP53, and PTEN mutations, negative for MGMT promoter methylation).

**Figure 3:**
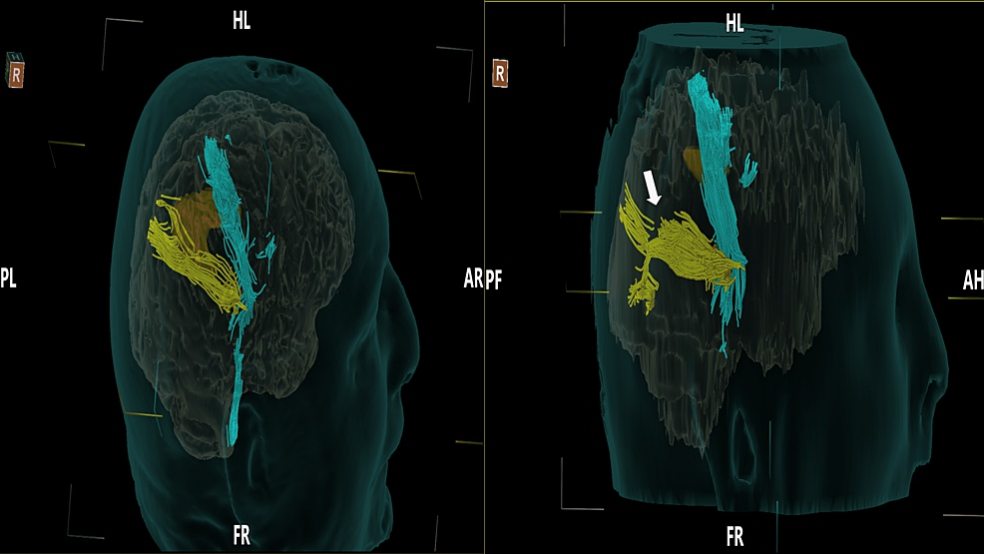
Pre (left) and postoperative (right) diffusion tensor tractography scan of the intracranial lesion (orange) following surgery The tumor has been significantly debulked with a small residual tumor embedded in the cortical spinal tract. Tract damage is visualized in the superior posterior optic radiation (white arrow). (Light blue: Corticospinal tract, Yellow: Optic pathway). HL - Head left, PL - posterior left, AR - anterior right, FR - foot right, PF - posterior foot, AH - anterior head.

Following the craniotomy, pre and postoperative tractography scans were used to evaluate quantitative comparisons of the white matter tracts. The preoperative tumor volume was found to be 10.4 cc while the postoperative tumor volume was 2.7 cc. Localized damage was visualized in the superior posterior aspect of the optic radiations (Figure 3). Additional metrics related to this pathway are discussed later in the paper.

For the corticospinal tract, the postoperative FA, GA, tract count, and tract volume metrics were found to significantly increase (p<0.0001) as a result of less tumor mass effect, which resulted in less compression of the tracts, more tract organization, and uniformity. MD, RD, and AD were found to decrease (p<0.0001) postoperatively, reflecting less degenerative insult to the myelin of the tracts (Table 1).

**Table 1:** Pre and postoperative quantitative metrics showing postoperative improvements of the FA, GA, tract count, and tract volume in the right corticospinal tract, with reductions in the MD, RD, and AD

Normalized preoperative versus postoperative tract values for the right corticospinal tract				
	Preop	Postop	Change	P value
Fractional Anisotropy (FA)	0.454905	0.507898	+12%	< .0001
Mean Diffusivity (MD)	0.514354	0.487508	-5%	< .0001
Radial Diffusivity (RD)	0.52429	0.486761	-7%	< .0001
Axial Diffusivity (AD)	0.502752	0.493575	-2%	< .0001
Geodesic Anisotropy (GA)	0.457338	0.505689	+11%	< .0001
Tract Count	0.407577	0.462667	+14%	< .0001
Tract Volume (cc, not normalized)	11.10448	19.83775	+79%	

For the optic radiations, similar postoperative improvements were found in the FA, GA, and tract count, as less tumor mass effect allowed for better uniformity of the optic radiations (p<0.0001). MD, RD, and AD values decreased postoperatively reflecting reduced degenerative insult to the tracts. The optic radiation volume showed a 4% decrease as a result of the partial transection of the tract seen in Figure 3 (Table 2).

**Table 2:** Pre and postoperative quantitative metrics showing postoperative improvements of the FA, tract count, and tract volume in the right optic radiations, with reductions in the MD, RD, AD, GA, and tract volume

Normalized preoperative versus postoperative tract values for the right optic radiations				
	Preop	Postop	Change	P value
Fractional Anisotropy (FA)	0.46859	0.51053	+9%	< .0001
Mean Diffusivity (MD)	0.52034	0.487164	-6%	< .0001
Radial Diffusivity (RD)	0.525601	0.483906	-8%	< .0001
Axial Diffusivity (AD)	0.50279	0.49284	-2%	< .0001
Geodesic Anisotropy (GA)	0.465362	0.510065	+10%	< .0001
Tract Count	0.528681	0.555406	+5%	< .0001
Tract Volume (cc, not normalized)	9.96857	9.533631	-4%	

## Discussion

Minimally invasive techniques are often used in cranial surgery in order to limit the amount of damage to eloquent white matter tracts. Diffusion tensor tractography has provided a means for surgeons to qualitatively evaluate white matter tracts to assist with surgical planning, however, with improving tractography software, extracting quantitative data from DTT and using it to assess WMT integrity is becoming more feasible. This study proposes a technique to evaluate novel quantitative features of an emerging DTI tractography technology and provides the first quantitative perioperative analysis from cranial tumor surgery.

Goals of DTI tractography

For tumor surgery, the goal of the operation is to maximize the tumor resection while minimizing damage to the normal brain tissue in an effort to extend patient survival, maintain viable functional status, and reduce the chance of recurrence [13]. DTI can assist with surgical planning by identifying eloquent white matter tracts that may surround a lesion requiring resection [14-18]. Qualitative DTI data, evaluating the white matter tract locations, are important for surgical planning; however, understanding the quantitative data generated from DTI may provide a means of correlating pre and postoperative neurologic deficits based on the quantitative assessments of tract distortion and integrity. For this case, the patient was found to have improvements in the FA and GA with decreased MD, RD, and AD likely from the reduced tumor mass effect as a result of tumor debulking. This translated into a motor improvement on the patient’s clinical strength exam. Although this case is too small of a sample size to draw any conclusions between the quantitative values and clinical presentation, future studies evaluating these relationships may present a promising means of evaluating neurosurgical patients.

Quantitative tractography measurements

Clinical studies have utilized fractional anisotropy (FA), radial diffusivity (RD), and mean diffusivity (MD) as ways to quantify DTT but never in the operative setting [4]. DTI measures the direction of water diffusion in every image voxel. This measurement consists of three eigenvectors and three eigenvalues that determine the magnitude and direction of water diffusion in each specific voxel [19]. Fractional anisotropy is a value that describes the anisotropy of the diffusion process, or the degree to which diffusion is restricted in a particular direction, often reflecting the axonal diameter, fiber density, and myelination [4,19]. Geodesic anisotropy measures the distance between a tensor to the closest isotropic tensor, can give a sense of space amongst a specific group of tract fibers, and can be indicative of mass effect on the tract [20]. Mean diffusivity is a measurement of the diffusion properties of a specific voxel, accounting for the three orthogonal directions in space. Radial diffusivity produces a perpendicular diffusivity from measures of two small axes tensors [4]. Axial diffusivity reflects the diffusion along the axon. Increases in these values can implicate pathologic degeneration of the nerve axon or demyelination of the white matter tracts and provides additional quantitative data for assessing DTI [5-6]. Interpretations of these values help estimate membrane integrity, axonal density, and myelination [4].

Prior studies have used various metrics to make quantitative characterizations of white matter tracts for particular neurologic conditions. Migliaccio et al. used MD, FA, parallel and perpendicular diffusivity, as well as streamlines (a measurement of tract volume) to evaluate the degree of neurodegenerations in patients with posterior cortical atrophy [21]. Ciccarelli et al. [22] looked at volume and FA in white matter tracts using fast marching tractography while Ding et al. [23] looked at torsion, curvature, parallel diffusivity, and perpendicular diffusivity of fiber tracts. Streamtubes, a metric describing a uniformed region of flow, have been evaluated for length and number to describe white matter axonal injury [19,24].

Despite these prior quantitative characterizations of the white matter tracts, no operative neurosurgical relevant application of quantitative tractography had previously been published until this paper. Our study attempts to report a novel technique that utilizes quantitative metrics to assess white matter tract integrity in the perioperative setting. By establishing a quantitative means to compare pre and postoperative imaging or even imaging between different patients, this paper establishes the foundation for correlating quantitative data to white matter tract integrity. This correlation may serve to help evaluate different aspects of neurosurgery, such as evaluating the collateral WMT damage to the brain as a result of various surgical techniques, or it may provide a better understanding of a patient’s neurological presentation based on the quantitative measures of associated WMTs. In this case presentation, the patient regained full strength with improvements in the metrics of this corticospinal quantitative data while his visual fields remained intact postoperatively, despite the changes to the quantitative optic radiations data. Further work will need to be done to associate these quantitative metric changes with clinical neurologic presentations, but this presents a promising opportunity for evaluating patients.

Limitations with diffusion tensor tractography

The potential of DTT for quantitative metrics also comes with limitations. Algorithms for DTT are often based on the known anatomy of healthy human brains, which may not correlate well with a pathologic brain and could alter the tractography [25-26]. There is no gold standard, and various algorithms exist for DTT [3]. Traditionally, DTI requires all particles involved to have identical behavior with a finite variance of the particles’ distribution of displacements [27]. Alterations of the anatomy, from edema or mass effect, can lead to poor MR signal or incorrectly calculated white matter tracts if the particle of a specific tract is distorted beyond known variations. DTT, a measurement of fiber tract direction based on voxel-averaged quantity, works well when the fiber tract anisotropy is uniform, but in areas where there is a non-uniform direction of the fiber tracts, DTT can have difficulty identifying the actual trajectory of the fiber tract [3]. Tracts with increased damage or low fractionated anisotropy could be missed altogether while crossing fibers can also present a degree of ambiguity in tractography [19,21].

In addition, the quality of the DTI acquisition can affect the tractography yield [19]. Subject movement during an MRI scan could alter the DTI metrics and would affect the reproducibility of data acquisition. This would warrant normalization techniques as was done in this study. DTI is also vulnerable to significant artifacts that can impair the veracity of the obtained scan [28]. Artifacts from foreign implants, such as deep brain stimulation, can also alter the imaging of DTI [4]. Intracranial pathology can present issues relating to adequate data collection with DTI. With tumor infiltration and peritumoral edema, the white matter tractography can be difficult to calculate as the diffusion trajectories of fluid in the tracts are greatly altered [29]. Vasogenic edema has also been shown to alter the anisotropy of the fiber tract to an extent where detecting accurate diffusion directionality to depict fiber tracts may not be possible due to the significant change in the transverse direction of the nerve fiber as a result of neuronal swelling [3]. In addition, ischemic white matter injury has been shown to have reduced anisotropy and increased diffusivity [30].

Comparing DTT scans can also present challenges. When trying to compare the tracts of different individuals, baseline white matter tract lengths and sizes vary from person to person [19]. Correia et al. proposed normalized metrics based on intracranial volume to try to remedy this issue [19]. In our study, scans from the same individual were compared, and normalizations were made to reduce the impact of motion artifacts using the contralateral tract which was found to have normal anatomy.

Despite the limitations of DTI, the continued improvement and optimization of fiber-tracking algorithm DTI tractography has begun to address many of these obstacles in order to depict a more reliable tractography output that can be used for quantitative measures.

## Conclusions

Diffusion tensor tractography has been previously shown to provide qualitative data to assist with surgical planning but appears to have the capacity to provide quantitative data that may provide comparative metrics to evaluate the white matter tract integrity in the perioperative setting. By quantitatively evaluating a multitude of WMT metrics, DTT has the potential of providing a new means of optimizing surgical techniques and the understanding the neurologic condition through quantitative measures.

## References

[1] Chen X, Weigel D, Ganslandt O, Buchfelder M, Nimsky C (2007). Diffusion tensor imaging and white matter tractography in patients with brainstem lesions. Acta Neurochir (Wien).

[2] Coenen VA, Schlaepfer TE, Allert N, Mädler B (2012). Diffusion tensor imaging and neuromodulation: DTI as key technology for deep brain stimulation. Int Rev Neurobiol.

[3] Yu CS, Li KC, Xuan Y, Ji XM, Qin W (2005). Diffusion tensor tractography in patients with cerebral tumors: a helpful technique for neurosurgical planning and postoperative assessment. Eur J Radiol.

[4] Muller J, Alizadeh M, Li L (2020). Feasibility of diffusion and probabilistic white matter analysis in patients implanted with a deep brain stimulator. Neuroimage Clin.

[5] Feldman HM, Yeatman JD, Lee ES, Barde LH, Gaman-Bean S (2010). Diffusion tensor imaging: a review for pediatric researchers and clinicians. J Dev Behav Pediatr.

[6] Vaillancourt DE, Spraker MB, Prodoehl J (2009). High-resolution diffusion tensor imaging in the substantia nigra of de novo Parkinson disease. Neurology.

[7] Clark CA, Barrick TR, Murphy MM, Bell BA (2003). White matter fiber tracking in patients with space-occupying lesions of the brain: a new technique for neurosurgical planning?. Neuroimage.

[8] Field AS, Alexander AL, Wu YC, Hasan KM, Witwer B, Badie B (2004). Diffusion tensor eigenvector directional color imaging patterns in the evaluation of cerebral white matter tracts altered by tumor. J Magn Reson Imaging.

[9] Gauvain KM, McKinstry RC, Mukherjee P, Perry A, Neil JJ, Kaufman BA, Hayashi RJ (2001). Evaluating pediatric brain tumor cellularity with diffusion-tensor imaging. AJR Am J Roentgenol.

[10] Guo AC, Cummings TJ, Dash RC, Provenzale JM (2002). Lymphomas and high-grade astrocytomas: comparison of water diffusibility and histologic characteristics. Radiology.

[11] Sinha S, Bastin ME, Whittle IR, Wardlaw JM (2002). Diffusion tensor MR imaging of high-grade cerebral gliomas. AJNR Am J Neuroradiol.

[12] Witwer BP, Moftakhar R, Hasan KM (2002). Diffusion-tensor imaging of white matter tracts in patients with cerebral neoplasm. J Neurosurg.

[13] Ammirati M, Vick N, Liao YL, Ciric I, Mikhael M (1987). Effect of the extent of surgical resection on survival and quality of life in patients with supratentorial glioblastomas and anaplastic astrocytomas. Neurosurgery.

[14] D'Andrea G, Familiari P, Di Lauro A, Angelini A, Sessa G (2016). Safe resection of gliomas of the dominant angular gyrus availing of preoperative FMRI and intraoperative DTI: preliminary series and surgical technique. World Neurosurg.

[15] Kis D, Máté A, Kincses ZT, Vörös E, Barzó P (2014). The role of probabilistic tractography in the surgical treatment of thalamic gliomas. Neurosurgery.

[16] Hayashi Y, Kinoshita M, Nakada M, Hamada J (2012). Correlation between language function and the left arcuate fasciculus detected by diffusion tensor imaging tractography after brain tumor surgery. J Neurosurg.

[17] Duc NM (2020). The role of diffusion tensor imaging metrics in the discrimination between cerebellar medulloblastoma and brainstem glioma. Pediatr Blood Cancer.

[18] Minh Duc N (2021). The performance of diffusion tensor imaging parameters for the distinction between medulloblastoma and pilocytic astrocytoma. Minerva Pediatr (Torino).

[19] Correia S, Lee SY, Voorn T (2008). Quantitative tractography metrics of white matter integrity in diffusion-tensor MRI. Neuroimage.

[20] Lee AD, Leporé N, Barysheva M (2008). Comparison of fractional and geodesic anisotropy in diffusion tensor images of 90 monozygotic and dizygotic twins. Proc IEEE Int Symp Biomed Imaging.

[21] Migliaccio R, Agosta F, Toba MN (2012). Brain networks in posterior cortical atrophy: a single case tractography study and literature review. Cortex.

[22] Ciccarelli O, Parker GJM, Toosy AT (2003). From diffusion tractography to quantitative white matter tract measures: a reproducibility study. Neuroimage.

[23] Ding Z, Gore JC, Anderson AW (2003). Classification and quantification of neuronal fiber pathways using diffusion tensor MRI. Magn Reson Med.

[24] Jiang H, van Zijl PC, Kim J, Pearlson GD, Mori S (2006). DtiStudio: resource program for diffusion tensor computation and fiber bundle tracking. Comput Methods Programs Biomed.

[25] Mamata H, Mamata Y, Westin CF, Shenton ME, Kikinis R, Jolesz FA, Maier SE (2002). High-resolution line scan diffusion tensor MR imaging of white matter fiber tract anatomy. AJNR Am J Neuroradiol.

[26] Catani M, Howard RJ, Pajevic S, Jones DK (2002). Virtual in vivo interactive dissection of white matter fasciculi in the human brain. Neuroimage.

[27] Ye AQ, Hubbard Cristinacce PL, Zhou FL, Yin Z, Parker GJ, Magin RL (2014). Diffusion tensor MRI phantom exhibits anomalous diffusion. Annu Int Conf IEEE Eng Med Biol Soc.

[28] Liu B, Zhu T, Zhong J (2015). Comparison of quality control software tools for diffusion tensor imaging. Magn Reson Imaging.

[29] Price SJ, Peña A, Burnet NG (2004). Tissue signature characterisation of diffusion tensor abnormalities in cerebral gliomas. Eur Radiol.

[30] Jones DK, Lythgoe D, Horsfield MA, Simmons A, Williams SC, Markus HS (1999). Characterization of white matter damage in ischemic leukoaraiosis with diffusion tensor MRI. Stroke.

